# Impact of Tremor on Patients With Early Stage Parkinson's Disease

**DOI:** 10.3389/fneur.2018.00628

**Published:** 2018-08-03

**Authors:** Lauren E. Heusinkveld, Mallory L. Hacker, Maxim Turchan, Thomas L. Davis, David Charles

**Affiliations:** ^1^Laboratory of Molecular Immunology, National Institute of Allergy and Infectious Diseases, National Institutes of Health, Bethesda, MD, United States; ^2^Department of Neurology, Vanderbilt University Medical Center, Nashville, TN, United States

**Keywords:** Parkinson's disease, deep brain stimulation, tremor, patient perspectives, tremor management

## Abstract

Tremor is one of the most visible features of Parkinson's disease (PD), and the majority of PD patients experience tremor during the course of the disease. However, the distress caused by this cardinal motor feature for patients early in the course of their PD is commonly underappreciated. People living with early stage PD often experience intense embarrassment and difficulties due to their tremor that limit social interactions, and tremor frequently interferes with the ability to perform activities of daily living and simple tasks at home and work. Although tremor is primarily managed with medications, both tremor response and satisfaction with medical therapy are highly variable. This review offers an overview of reports of the patient experience of tremor in early stage PD and current management options for this cardinal motor feature.

## Introduction

Parkinson's disease (PD) is a movement disorder that is the second most common neurodegenerative disease. Over one million Americans currently live with PD, and ~60,000 patients are newly diagnosed each year (1). Furthermore, the incidence of PD is only expected to increase in the coming decades (2). Parkinson's disease is a heterogeneous condition involving both motor and non-motor features that contribute to diminishing quality of life for patients as the disease progresses. Although a myriad of signs and symptoms contribute to PD-related disability, early stage PD is largely characterized by cardinal motor symptoms, including bradykinesia, tremor, and rigidity (3).

Tremor is one of the most visible features of the disease, and rest tremor is often the first PD symptom noticed by patients (4). More than 75% of PD patients experience rest tremor at some point during the disease course (5–7), and ~60% of patients experience symptomatic tremor during action or movement (8–10). Although the near-ubiquity of tremor in early PD is beyond dispute, the distress caused by this cardinal motor symptom is commonly underappreciated (11). Tremor in early PD patients is currently managed pharmacologically. However, medications offer limited relief from tremor and may lead to disabling adverse effects.

The purpose of this review is to provide an overview of the patient experience and current management of tremor for patients with early stage PD. Early stage PD is frequently defined as Hoehn & Yahr Stage I (mild, unilateral motor symptoms) or II (bilateral motor symptoms without balance impairment) (12). However, there are no universally-accepted criteria for defining PD stages, and definitions vary across clinical trials and studies. For clarity, we have described the criteria used to define early stage PD for each study included in this review. To conduct the review, we searched PubMed and Google Scholar using combinations of the terms “early stage Parkinson's disease,” “early Parkinson's,” “tremor,” “patient perspective,” “therapy,” “cognitive behavioral therapy,” “support group,” and “survey.”

## Impact of tremor for patients with early stage parkinson's disease

Patients with early stage PD consistently rank tremor as highly important, even when asked to consider other diverse aspects of their condition. Tremor was cited as the most bothersome symptom in a survey of 75 PD patients with relatively mild symptom severity (median PDQ-39 summary index score = 20.3; estimated median modified Hoehn & Yahr stage = I to I.5) (13). Twenty-eight percent (21/75) of patients mentioned tremor in their open-ended response to the question, “Which two problems related to Parkinson's disease bother you most?”

In a separate PD patient survey, nearly one-third of patients with early stage PD ranked tremor as their most troubling symptom, and tremor was the second most highly ranked symptom among all early stage patients (14). In this study, early stage PD was defined as a symptom duration of <6 years, while a symptom duration equal to or exceeding 6 years was considered advanced stage disease. Politis et al. asked 265 patients to list and rank the three PD symptoms that most affected their quality of life in the preceding 6 months. Overall, tremor was listed in the top three most troubling symptoms by nearly half of early stage PD participants (42%). Furthermore, tremor was the only motor symptom in the top ten most bothersome symptoms among advanced stage survey participants (ranked fifth highest). This report suggests that as PD progresses and non-motor symptoms begin to dominate, tremor remains the only motor symptom patients consider distressing. Thus, tremor impacts quality of life for PD patients in the early stages and continues to be problematic throughout the course of the disease. It is worth noting that tremor for some patients may diminish in late-stage PD (15, 16), which could explain the discrepancy between tremor rankings among early- and late-stage PD patients.

The psychosocial impact of tremor for many PD patients is insidious and profound and goes well beyond a general annoyance (17). During an in-depth structured interview (17), patients and caregivers shared emotional and highly compelling stories of trying to disguise tremor during the early stages of PD by wearing clothes with pockets or hiding an affected hand behind one's back. They went further to express dreading the progression of their tremor during later stages of PD with remarks such as, “…as it changes to slavering and trembling in a corner, I will find that a horror.”

Parkinson's disease-related tremor is a source of significant social anxiety and embarrassment that negatively affects early stage patients' self-image, sense of security, and well-being (18, 19). In a survey of 103 PD patients (93.5% Hoehn & Yahr stage I or II), Louis et al. found that more than one-quarter agreed with following statements: “My tremor makes me feel negative about myself; I am embarrassed about my tremor; I am depressed because of my tremor; I worry about the future; I am nervous or anxious; I have difficulty concentrating because of my tremor” (18). In a separate survey of 100 PD patients, 36 indicated other people often comment on their tremor (19). These findings highlight the psychosocial impact tremor can have for patients with early stage PD.

Tremor also adversely affects daily living activities for patients. More than one-third of people with early stage PD identified tremor-related difficulty with numerous everyday tasks, which included writing, using a type-writer/computer, fixing small things, dressing, eating, and holding reading material (18). Given that the average age at PD onset is 55–65 years old (20, 21), many people are still employed when their tremor first becomes noticeable. Thus, it is not surprising that tremor was among the top three PD-related challenges patients reported facing at work (22). Parkinson's disease symptoms usually begin on the dominant-hand side (23), which could compound the disability associated with tremor in early stage PD. In the movement disorder patient survey reported by Louis et al., 17.6% of respondents with PD endorsed retiring early because of tremor-related difficulties, and 28.7% reported giving up hobbies due to their tremor (18). When asked to answer the following question in their own words: “What two problems related to tremor bother you most?” 28% of participants described tremors/shaking, which was the most frequently cited problem (13). Furthermore, PD patients reported spending an average of 5.5 waking hours with tremor each day (19). Parkinson's disease patients with the most severe tremor scores on the Unified Parkinson's Disease Rating Scale (UPDRS) experienced on average 7.9 waking hours per day with tremor.

These reports present the patient perspective on the distressing nature of tremor in early stage PD. People living with early stage PD often experience intense embarrassment and difficulties due to their tremor that limit social interactions, and tremor for many patients interferes with their ability to perform activities of daily living and simple tasks at home and work. Unfortunately, these psychosocial and physical impairments due to tremor persist even as PD progresses and non-motor symptoms begin to dominate.

## Management of tremor: levodopa and deep brain stimulation

Medication for tremor in PD is initiated when symptoms become distressing or embarrassing in social settings, and levodopa and dopamine agonists are typically prescribed (3, 24). Antiparkinsonian medications result in a reduction in the amplitude of tremor but not frequency (25). Although early PD symptoms are generally considered well-controlled by antiparkinsonian medications, tremor response is less robust compared to other dopaminergic symptoms, such as bradykinesia and rigidity (26). Parkinson's disease patients, many of whom had “mild on average” symptom severity (13), echoed these limitations of their current medication options through free-text responses to the following question: “What kind of assistance might help you cope or solve [problems related to PD]?”

“*I look forward to medication to diminish my tremor symptoms and hope to delay progression of the disease*.”

“*I try to accomplish things when I know that my medication will be at its peak time*.”

Levodopa is considered the gold standard for treating PD motor symptoms (27–29), yet tremor response to levodopa is highly variable among PD patients throughout the disease course. Although Parkinsonian tremor initially responds to dopaminergic agents in a majority of patients, it may become resistant to medications as PD progresses (29). Tremor control often comes at the expense of requiring increasing doses of medications (30), and treatment consistency becomes difficult to maintain due to the need for frequent adjustments to the medication regimen (29). Furthermore, the response duration to levodopa shortens over time, which leads to motor fluctuations that must be counteracted by higher and more frequent doses (31). In addition, cognitive stress both increases the intensity of PD-related tremor and reduces the tremor-attenuating effect of levodopa, which further complicates tremor management (32). Non-dopaminergic circuits may contribute to Parkinsonion tremor (32) and constitute an additional therapeutic target for tremor. Other medications for managing tremor are less effective and/or associated with intolerable side effects (24, 33). Thus, treating tremor pharmacologically remains challenging.

Just as treatment response is variable, so too is patient satisfaction with PD treatments. Davidson et al. (34) identified discrepancies between subjective patient responses and objective physician ratings. One-third of patients in a prospective study who showed no improvement on the motor section of the UPDRS at their first follow-up visit rated their improvement as “moderate” or better at a second follow-up, while 29% of those with more than 50% improvement on the motor UPDRS stated they had no or slight improvement (34). In this study, “no improvement” was defined as unchanged or worse UPDRS motor section score since the previous visit. The authors suggested that patients who acknowledged little motor improvement may have been disappointed by inadequate attenuation of their tremor. Future studies should investigate whether early stage patients and physicians perceive the tremor response to medication differently.

Deep brain stimulation (DBS) is an adjunctive therapy that provides excellent tremor control (35–37). Patients reflect dramatic improvement in their lives following DBS surgery due to reduction or abolition of tremor (11). Tremor before DBS surgery is described as pervasive and inescapable (11). Parkinson's disease patients experience intense psychological sequelae as a result of prolonged intractable tremor prior to surgery, including feelings of embarrassment and shame. In a qualitative study of 42 advanced PD patients who received DBS, tremor was considered the most significant problem before surgery for 60% (25/42) (11). After receiving DBS, patients experienced improvement in numerous aspects of daily life, including more satisfying relationships, reduced visibility of PD, improved performance at work, and greater independence. According to a survey examining the impact of DBS therapy on routine driving for PD patients, tremor and akinesia were the two PD symptoms most improved by DBS (38). DBS plus medication may attenuate resting and postural tremor frequency in addition to amplitude, unlike medication alone which only reduces amplitude (39). For many patients, DBS provides welcome relief from the distress caused by their tremor in earlier stages of PD. Currently, DBS is approved by the FDA for PD patients with disease duration of at least 4 years and recent onset of motor fluctuations.

Whether DBS therapy could be applied in very early stage PD is still under investigation, with a pilot trial complete (40) and a pivotal trial in development. Interestingly, a recent retrospective analysis of the pilot safety and tolerability clinical trial of DBS in a very early stage PD population suggests DBS may slow the progression of rest tremor (41). Thirty early stage PD patients (Hoehn & Yahr II off medication, treated with medications 0.5–4 years) were randomized to DBS + optimal drug therapy (ODT) or ODT alone. Analysis of individual motor items of the single-blind UPDRS-III collected after a 7-day therapeutic washout of all medications and stimulation suggests that early DBS may slow the progression of rest tremor. Patients also reflected the impact early DBS had on their tremor, with 46% (6/13) of respondents to an end-of-trial survey spontaneously indicating that tremor improvement was the greatest benefit of undergoing DBS surgery. While intriguing, these results require further investigation in a larger prospective cohort.

Although DBS offers robust tremor management for mid- and advanced stage patients, therapies for tremor control in early stage PD are currently limited to pharmacological options, primarily levodopa. Levodopa offers variable relief from symptoms and is associated with significant side-effects that often preclude lifelong use, including irreversible levodopa-induced dyskinesias (LIDs) in most patients (42). Tremor control remains a source of concern for early stage PD patients, and future studies should explore alternative treatments for tremor in this population.

## Non-medical strategies for coping with tremor

Given the profound psychosocial implications of tremor for patients with early stage PD, non-medical strategies for coping with tremor should be considered. Several participants in Uebelacker et al.'s survey indicated peer support groups are helpful (13). A cross-sectional study reported that attending a support group is associated with better quality of life and reduced depression and anxiety for PD patients (43). Cognitive behavioral therapy (CBT) has been investigated for addressing depression and anxiety in PD in several clinical trials with promising results (44–48). A new model of CBT has been proposed to adapt cognitive behavioral maintenance models to patients with PD, and a case example illustrates how it could be applied for a patient who is struggling to cope with tremor (49). The efficacy of these psychosocial interventions for early stage PD patients with bothersome tremor is unknown. A randomized, open-label, parallel group, controlled efficacy trial did not support the use of low-dose occupational therapy in patients with mild to moderate PD in the UK (67% Hoehn and Yahr ≤ II) (50, 51).

## Conclusion

Tremor is a cardinal motor feature of PD that many patients consider to be distressing, yet the impact of tremor on early stage PD patients remains largely underappreciated (11). Multiple features of daily life are negatively impacted, including personal and social relationships, self-image, and overall sense of well-being, in addition to activities of daily living and other tasks at home and work. Together, these findings highlight that there is considerable room for improvement in the management of tremor in early stage PD. Future research is needed to investigate ways to support PD patients for whom tremor causes significant distress and explore alternative treatments for tremor in early stage PD. The FDA has approved (IDE G050016) a phase III, double-blind, placebo controlled clinical trial of subthalamic nucleus DBS in 280 very early stage PD subjects across 18 US centers. The FDA has further approved a key secondary analysis for this trial to be focused on determining if the therapy slows the progression of rest tremor when applied during early stage disease.

## Author contributions

DC and MH contributed to the conception of the work. LH, MH, and MT conducted the literature review. LH wrote the first draft of the manuscript. MH and DC contributed to the writing of all sections. MT and TD revised the manuscript critically for intellectual content. All authors contributed to manuscript revision and read and approved the submitted version.

### Conflict of interest statement

Vanderbilt University receives income from grants and contracts with Allergan, Ipsen, Lundbeck, Merz, Medtronic, and USWorldMeds for research or educational programs led by DC. DC receives income from Allergan, Alliance for Patient Access, Ipsen, Medtronic, and Revance for consulting services. The remaining authors declare that the research was conducted in the absence of any commercial or financial relationships that could be construed as a potential conflict of interest.
